# Potassium-Based Solid Sorbents for CO_2_ Adsorption: Key Role of Interconnected Pores

**DOI:** 10.3390/nano14221838

**Published:** 2024-11-17

**Authors:** Yuan Zhao, Jiangbo Huo, Xuefei Wang, Shunwei Ma

**Affiliations:** 1 Tianjin College, University of Science and Technology Beijing, Tianjin 301830, China; zhaoyuan01277@sina.com (Y.Z.);; 2 Tianjin Key Laboratory of Aquatic Science and Technology, School of Environmental and Municipal Engineering, Tianjin Chengjian University, Tianjin 300384, China

**Keywords:** CO_2_, potassium carbonate, adsorbent, aluminum oxide

## Abstract

Industrial CO_2_ emissions contribute to pollution and greenhouse effects, highlighting the importance of carbon capture. Potassium carbonate (K_2_CO_3_) is an effective CO_2_ absorbent, yet its liquid-phase absorption faces issues like diffusion resistance and corrosion risks. In this work, the solid adsorbents were developed with K_2_CO_3_ immobilized on the selected porous supports. Al_2_O_3_ had an optimum CO_2_ adsorption capacity of 0.82 mmol g^−1^. After further optimization of its pore structure, the self-prepared support Al_2_O_3_-2, which has an average pore diameter of 11.89 nm and a pore volume of 0.59 cm^3^ g^−1^, achieved a maximum CO_2_ adsorption capacity of 1.12 mmol g^−1^ following K_2_CO_3_ impregnation. Additionally, the relationship between support structure and CO_2_ adsorption efficiency was also analyzed. The connectivity of the pores and the large pore diameter of the support may play a key role in enhancing CO_2_ adsorption performance. During 10 cycles of testing, the K_2_CO_3_-based adsorbents demonstrated consistent high CO_2_ adsorption capacity with negligible degradation.

## 1. Introduction

The increasing concentration of CO_2_ in flue gas from coal-fired power plants, a critical driver of the greenhouse effect, has led to the recognition that Carbon Capture and Storage (CCS) technology, particularly post-combustion capture, is regarded as a promising solution for the reduction of carbon dioxide emissions [1,2,3,4,5,6]. Although the liquid amine absorption method has been widely utilized in post-combustion capture, it still faces several challenges, including corrosiveness to containment vessels, potential amine volatility leading to environmental contamination, and significant mass transfer resistance within the liquid phase [7,8,9,10]. Recently, the incorporation of organic amines into porous supports has been extensively studied by many researchers [11,12,13,14,15,16,17,18,19,20,21,22,23,24]. Solid amine adsorbents, utilizing materials with high porosity such as polymers [11,12,13], carbon materials (e.g., carbon nanotube [14,15], activated carbon [16,17], and mesoporous carbon [18]), and mesoporous molecular sieves (e.g., SBA-15 [19,20,21], MCM-41 [22,23], and KIT-6 [24]), have been a focal point of research. These adsorbents incorporate various amine groups (e.g., primary, secondary, and tertiary amines) to enhance CO_2_ capture capabilities [25]. However, despite their high adsorption capacity, the surface utilization rate of amine groups is still quite low. This is mainly because the organic amine has high viscosity and large molecular diameter, making it hard to penetrate evenly in the pores, which can lead to aggregation, pore blockage, and ultimately fewer active sites on the surface. Furthermore, there is significant amine loss during the adsorption–desorption cycles due to the facts that amines are inherently volatile organic compounds. Thus, it is necessary to develop innovative technologies or materials to overcome the current limitations in efficiency and cost.

In recent years, numerous studies have reported the use of potassium carbonate as an active component impregnated onto support materials to fabricate K_2_CO_3_-based adsorbents, which serve as a substitute for amine-based adsorbents for CO_2_ capture [26,27,28]. Alkali carbonate-based adsorbents exhibit superior stability and are free from issues of secondary environmental pollution. They are more difficult to decompose, even when the temperature is increased to 800 °C. Generally, a temperature of 350 °C is adequate to fully regenerate the adsorbent. The inherent alkalinity of potassium carbonate confers a significantly enhanced CO_2_ adsorption capacity upon the adsorbent, particularly effective for the capture of low-concentration CO_2_, such as the 10–15% found in flue gasses. Current research on K_2_CO_3_-based adsorbents predominantly focuses on the in-depth exploration of the underlying reaction mechanisms and the selection of appropriate support materials [29,30,31,32,33,34,35,36,37,38,39,40,41,42,43]. The carbonation reaction induced by K_2_CO_3_-based sorbents was found to consist of two steps: first, the hydration reaction takes place, and then the produced KHCO_3_ reacts rapidly. During the CO_2_ sorption process, the carbonation reaction coexists with the adsorption process, indicating that both chemical and physical adsorption occur [28]. The support material in K_2_CO_3_-based sorbents facilitates not only physical CO_2_ adsorption but also the dispersion of alkaline active sites. Consequently, the choice of support material and the optimization of its pore structure are crucial for enhancing the CO_2_ adsorption performance of the sorbents.

Activated carbon was first considered as a superior support material due to its high surface area, abundant porosity, and controllable pore structure. It was reported that the K_2_CO_3_/AC sorbent exhibits high CO_2_ capture capacities and rapid carbonation reaction rates [28,29,30]. Zeolites have also been widely investigated because they possess well-defined pore structures that can accommodate both the immobilized alkali metal carbonates and the adsorbed CO_2_ [31,32,33,34,35,36,37,38]. In addition, activated alumina, with its high porosity, large surface area, and diverse pore channels, also has great potential for use as a support material [39,40,41,42,43]. Zhao et al. [40,41,42,43] have conducted extensive research on the supports for K_2_CO_3_-based sorbents. They impregnated potassium carbonate onto several supports and compared the conversion rates using thermogravimetric analysis (TGA) and a bubbling fluidized-bed reactor. However, the pore structure of the support materials requires additional refinement to optimize performance. There is an imperative to elucidate the correlation between the pore structural attributes and CO_2_ adsorption efficacy, which is pivotal for informing the strategic design of advanced support materials. In this study, the performance of adsorbents prepared with various support materials was systematically compared, and the pore structure was further improved by strategically selecting the most suitable support. Additionally, the research endeavored to delineate the principal factors that influence CO_2_ adsorption capacity.

## 2. Materials and Methods

### 2.1. Acquisition of the Support Materials

The porous solid materials, including ZSM-5, zeolite 5A, zeolite β, zeolite NaY, and MCM-41, served as supports and were obtained from Nanjing XFNANO Materials Tech Co., Ltd., Nanjing, China. The activated carbons, namely, CSAC and CAC, were purchased from Kecheng Activated Carbon Co., Ltd., Beijing, China. Aluminum oxide (Al_2_O_3_) was supplied by J&K Scientific. Moreover, two additional aluminum oxide supports were self-synthesized using the following procedures:

Scheme 1 [44]: In the preparation of Al_2_O_3_-1, 2.38 g of sodium aluminate, 20.23 g of urea, and 1 g of F127 were combined and dissolved in 70 mL of distilled water under vigorous stirring to form a homogeneous solution. This solution was subsequently transferred to a 100 mL Teflon-lined stainless steel autoclave, sealed, and subjected to hydrothermal treatment at 140 °C for 24 h. After that, the autoclave was cooled to ambient temperature, and then a white precipitate was formed. The resulting white precipitate was isolated from the supernatant and extensively washed with deionized water followed by ethanol in a sequential manner. The precipitate was then dried in a vacuum oven at 80 °C for 12 h. Finally, the dried powder was calcined at 450 °C for 4 h in static air to yield the desired γ-Al_2_O_3_ product.

Scheme 2 [45]: For the synthesis of Al_2_O_3_-2, a mixture of Al(NO_3_)_3_·9H_2_O (0.014 mol) and CO(NH_2_)_2_ (0.028 mol, 0.021 mol, or 0.056 mol) was dissolved in 70 mL of distilled water and stirred vigorously for 30 min to form a clear solution. The solution was then poured into a 100 mL Teflon-lined autoclave and heated at 180 °C (160 °C or 200 °C) for 4 h (3 h or 5 h), followed by natural cooling to room temperature. The resulting white precipitate was collected by vacuum filtration, washed sequentially with distilled water and anhydrous alcohol, and dried at 80 °C for 12 h in a vacuum oven. The boehmite precursor was calcined at 550 °C for 4 h, with a heating rate of 4 °C min^−1^, to yield the final γ-Al_2_O_3_ product.

All the reagents used in the two schemes were of analytical grade without any further purification.

### 2.2. Preparation of K_2_CO_3_-Based Adsorbents

To prepare K_2_CO_3_-based sorbents, designated as x K_2_CO_3_/support, where x represents the weight percentage of K_2_CO_3_ in the adsorbents, a given amount of K_2_CO_3_ was first dissolved in 40 mL of methanol with stirring for 30 min at room temperature. Then, 2 g of the supports was added into the above K_2_CO_3_ methanol solution. After that, it was mixed with a magnetic stirrer at 40 °C until most of the methanol evaporated. Finally, the resultant x K_2_CO_3_/support adsorbents were further dried at 80 °C for 12 h under vacuum.

### 2.3. Characterization of the Self-Synthesized Al_2_O_3_

X-ray diffraction (XRD) spectra were obtained by an X’Pert PRO diffractor (PANalytical, Almelo, Holland, Cu Kα, λ = 0.15406 nm, 40 kV, 40 mA). The micro-morphology of the Al_2_O_3_ supports was observed by a scanning electron microscope (SEM, Hitachi SU8010, Tokyo, Japan). Before the measurements, the samples were degassed at 100 °C for 12 h under vacuum. Nitrogen adsorption–desorption isotherms were measured at 77 K using a Tristar II 3020 analyzer (Micromeritics, Norcross, GA, USA). The Brunauer–Emmett–Teller (BET) method was utilized to calculate the specific surface area. The total pore volume and pore size distribution were derived from the isotherm desorption branches using the Barrett–Joyner–Halenda (BJH) model. A thermogravimetric analysis (TGA, Netzsch STA 449F5, Selby, Germany) of samples was performed in a highly pure N_2_ atmosphere at a flow rate of 70 mL/min. About 10 mg of the sample was heated at a constant rate of 10 °C/min from room temperature to 600 °C.

### 2.4. Evaluation for CO_2_ Adsorption over K_2_CO_3_-Based Adsorbents

**Figure 1:**
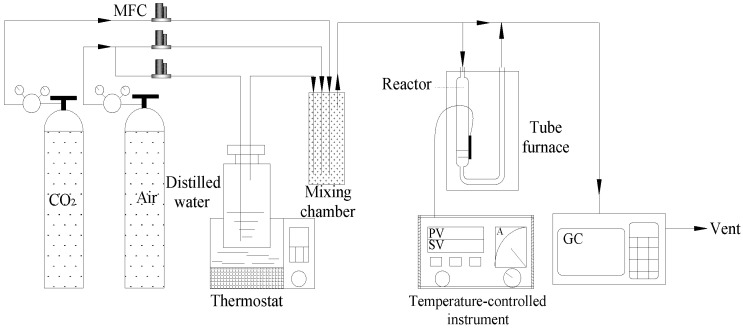
Schematic diagram of the experimental setup for CO_2_ adsorption assessment.

A fixed-bed flow sorption system, equipped with gas flow controllers and an integrated online gas chromatograph, was designed and constructed for the purpose of evaluating adsorbent performance in CO_2_ adsorption, as depicted in Figure 1. The process involved packing 2 g of adsorbent into a U-shaped quartz reactor, which was placed in a programmable furnace for precise temperature control. Prior to each measurement, the sample was heated to 100 °C in a highly pure Ar stream at the flow rate of 100 mL min^−1^ for 60 min to eliminate the physically adsorbed H_2_O and CO_2_, and then the sample was cooled to 25 °C. The gas stream was rapidly switched to the 10% CO_2_/air with 30% relative humidity (RH) at the desired flow rate of 10 mL min^−1^. The moisture was produced by bubbling air into water, and the relative humidity was measured using a hygrometer. The flow rate of the gas was controlled by electronic flow control instruments. The concentrations of CO_2_ at the inlet and outlet of the reactor were monitored by an online gas chromatograph (GC-7890II, Techcomp, Beijing, China) equipped with a methane converter, and the flame ionization detector was used. The sorption capacity of the adsorbent was calculated by the integration of the area above the breakthrough curve, and the integral equation is displayed in Equation (1)

(1) $$q_{s}=\frac{1}{W}\times [\int_{0}^{t}Q\times \frac{C_{0}-C}{1-C}d_{t}]\times \frac{T_{0}}{T}\times \frac{1}{V_{m}}$$

where *q*_s_ is the saturated adsorption capacity of CO_2_, mmol g^−1^. *W* is the weight of the adsorbent, g. *Q* is the gas flow rate mL min^−1^. *C*_0_ and *C* are the influent and effluent CO_2_ concentration, respectively, vol%. *t* denotes the adsorption time, min. *T*_0_ is 273K. *T* is the gas temperature, 273K. *V*_m_ is 22.4 mL mmol^−1^. *q*_s_ is defined as the adsorption capacity of CO_2_ when *C* is equal to *C*_0_; i.e., *C*/*C*_0_ is equal to 1.0.

## 3. Results and Discussion

### 3.1. CO_2_ Sorption Performances of Different K_2_CO_3_-Based Adsorbents

**Figure 2:**
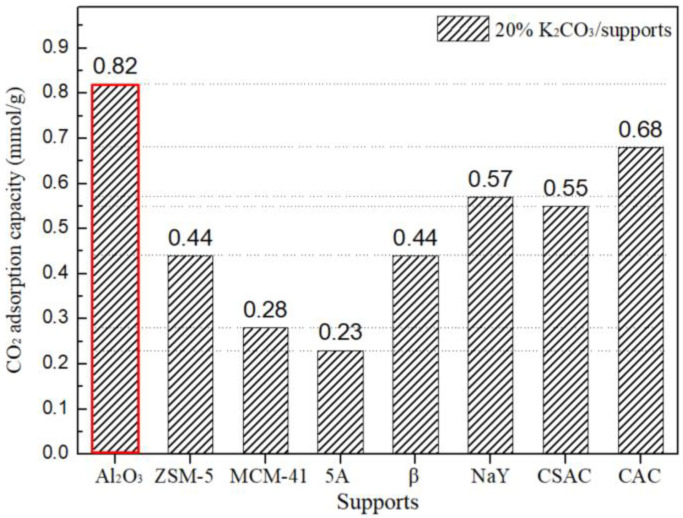
CO_2_ adsorption capacities of the 20% K_2_CO_3_/supports under the conditions: 10 vol% CO_2_ in air, 298 K adsorption temperature.

A series of K_2_CO_3_-based adsorbents were synthesized using the impregnation method, uniformly incorporating a 20% mass percentage of K_2_CO_3_ onto various support materials. The supports selected included aluminum oxide (Al_2_O_3_), coconut shell activated carbon (CSAC), coal-based activated carbon (CAC), and a series of molecular sieves, namely, zeolite ZSM-5, zeolite MCM-41, zeolite 5A, zeolite β, and zeolite NaY. The loading amount of K_2_CO_3_ was determined according to the literature [46,47], and the supports were chosen for their heterogeneous pore texture and abundant porosities. A comparison of the CO_2_ adsorption capacities for these adsorbents, each loaded with 20% K_2_CO_3_, was conducted under identical conditions, as depicted in Figure 2.

Porous solid materials are primarily characterized by the physical adsorption of CO_2_. Given that our study focuses on a low concentration of CO_2_ at 10%, the physical adsorption effect results in a low adsorption capacity, which can fall below the detection limit of gas chromatography. Consequently, the CO_2_ adsorption capacity of the supports is not depicted here. For the adsorbents with 20% K_2_CO_3_ loading, the 20% K_2_CO_3_/Al_2_O_3_ adsorbent exhibits a higher CO_2_ adsorption capacity compared to the other adsorbents. This superior performance may be attributed to the unique pore structure of Al_2_O_3_, which facilitates a homogeneous distribution of K_2_CO_3_ and promotes efficient contact between CO_2_ and the alkaline adsorption sites.

Due to its excellent performance, the pore structure and morphology of the Al_2_O_3_ support were further optimized by using different preparation conditions. Two types of Al_2_O_3_ were synthesized using different precursor solutions, as described in the experimental section. In Scheme 1, Al_2_O_3_-1 was formed by adding urea and F127 to sodium aluminate as the aluminum source. In Scheme 2, Al_2_O_3_-2 was produced by incorporating urea into a solution containing aluminum nitrate as the aluminum source. Both Al_2_O_3_-1 and Al_2_O_3_-2 were then subjected to impregnation with 20% K_2_CO_3_, resulting in CO_2_ adsorption capacities of 0.69 mmol g^−1^ and 1.12 mmol g^−1^, respectively.

**Figure 3:**
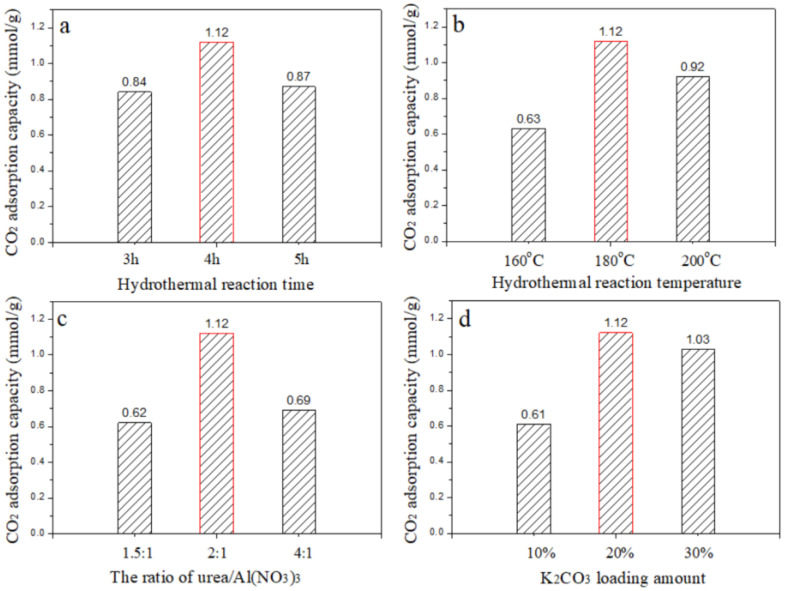
(**a**–**d**) CO_2_ adsorption capacities of 20% K_2_CO_3_/Al_2_O_3_-2 adsorbents by varying preparation conditions.

The 20% K_2_CO_3_/Al_2_O_3_-2 adsorbent exhibited a significantly higher CO_2_ adsorption capacity than 20% K_2_CO_3_/Al_2_O_3_-1. Thus, further adjustments were made to the Al_2_O_3_-2 support, including varying the hydrothermal reaction time, temperature, and the molar ratio of urea to aluminum nitrate nonahydrate (Al(NO_3_)_3_·9H_2_O) during the hydrothermal process. The obtained results are presented in Figure 3.

Figure 3a,b show the effects of hydrothermal reaction time and temperature on the preparation process, respectively. Clearly, either an excessively short reaction time or an undesirably low temperature can hinder the formation of Al_2_O_3_ crystals with perfection. Conversely, an excessively long reaction time or high temperature can damage the pore structure, affecting the CO_2_ adsorption performance of Al_2_O_3_ after K_2_CO_3_ loading. Figure 3c illustrates the optimization of the urea/Al(NO_3_)_3_ ratio. Proper amounts of urea and Al(NO_3_)_3_ are crucial for precise Al_2_O_3_ formation; excess amounts can lead to impurities, reducing the product’s purity and performance. Therefore, the optimal preparation conditions were determined to be a 2:1 molar ratio of urea to Al(NO_3_)_3_·9H_2_O in the precursor solution, with a hydrothermal reaction at 180 °C for 4 h.

Figure 3d exhibits the effect of varying the K_2_CO_3_ loading amount, indicating that the optimal loading is 20%. At a 10% loading, there are insufficient alkaline active sites on the adsorbent surface, resulting in lower CO_2_ adsorption capacity. Conversely, at a 30% loading, an excess of K_2_CO_3_ may block the pores, leading to the uneven dispersion of active sites, which in turn results in a lower utilization rate and a subsequent decrease in CO_2_ adsorption capacity.

Based on the optimal adsorption capacity attained, which was 1.12 mmol g^−1^, the utilization rate of K_2_CO_3_ was evaluated. Theoretical calculations, derived from the chemical reaction equation K_2_CO_3_ + H_2_O + CO_2_ ⇌ 2KHCO_3_, suggest that each gram of the adsorbent is capable of potentially adsorbing up to 1.45 mmol g^−1^ of CO_2_. As a result, the calculated maximum utilization rate for K_2_CO_3_ is approximately 77%.

### 3.2. Characterization of Self-Prepared Al_2_O_3_

**Figure 4:**
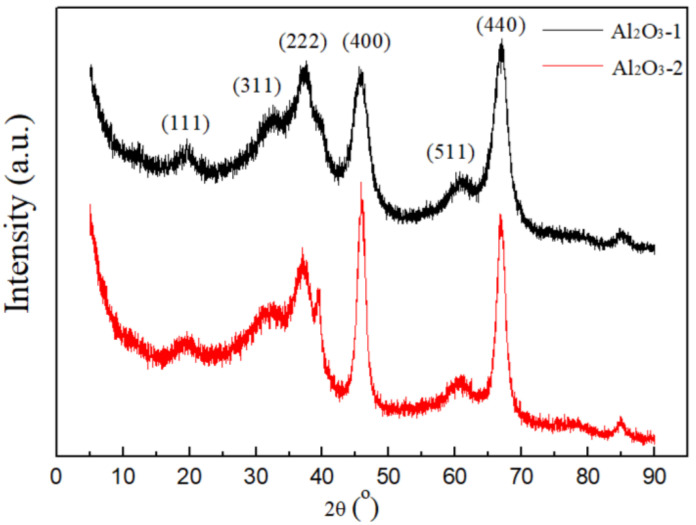
XRD patterns of Al_2_O_3_-1 and Al_2_O_3_-2.

The Al_2_O_3_ powders, synthesized using two different precursor solutions, have been subjected to comprehensive characterization and analysis. The X-ray diffraction (XRD) patterns for the Al_2_O_3_-1 and Al_2_O_3_-2 samples are presented in Figure 4. The patterns show that the reflective peaks observed in both samples are definitively attributed to cubic γ-Al_2_O_3_ [48], indicating a complete transformation achieved through the calcination process. In addition, the absence of any characteristic peaks from other crystalline impurities suggests that the samples exhibit an exceptionally high degree of purity. Notably, Al_2_O_3_-2 exhibits narrower diffraction peaks and relatively elevated peak intensities, indicating superior crystallinity and enhanced crystal quality. High crystallinity γ-Al_2_O_3_ may exhibit fewer defects and structural distortions, which is beneficial for improving the pore structure of the support and achieving a uniform distribution of surface active-sites [49].

**Figure 5:**
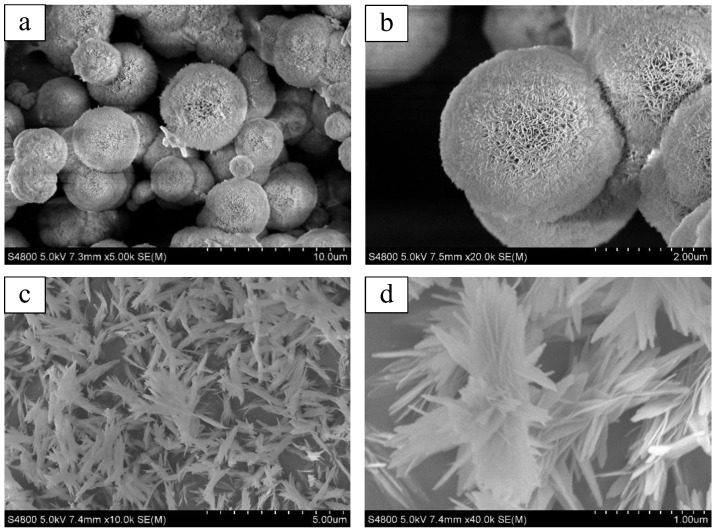
SEM images of Al_2_O_3_-1 (**a**,**b**) and Al_2_O_3_-2 (**c**,**d**).

The morphologies of Al_2_O_3_-1 and Al_2_O_3_-2 were examined using scanning electron microscopy (SEM). The spherical morphologies of Al_2_O_3_-1 are clearly observable in Figure 5a,b. It has been reported that hydrogen bonds between the surface of aluminum hydroxide (the precursor to γ-Al_2_O_3_) and the structure-directing agent molecules can reduce the free energy of the crystals, leading to the formation of low-dimensional nanosheets [50]. These nanosheets tend to aggregate to minimize the exposed area and thus reduce surface energy. Consequently, hierarchical porous γ-Al_2_O_3_ particles with a similarly spherical structure were formed through the directed self-assembly mediated by F127. The hierarchical porous structure not only accommodates K_2_CO_3_, providing basic active sites for CO_2_ adsorption, but also provides diffusion channels for CO_2_. Figure 5c depicts a low-magnification SEM image of Al_2_O_3_-2, showing that the sample is composed of well-dispersed spindle-like aggregates, and the irregular agglomerates are almost negligible, indicating the high quality and purity of the spindle-like aggregates. Figure 5d, a high-magnification SEM image, reveals that the three-dimensional spindle-like particles consist of well-aligned nanoplates with spindle-like edges and rough surfaces.

Obviously, compared to the spherical structure of Al_2_O_3_-1, the three-dimensional spindle-shaped pore structure in Al_2_O_3_-2 may be more conducive to the impregnation and uniform dispersion of K_2_CO_3_. It is likely that the spindle-like porous structure not only facilitates the entry of K_2_CO_3_ but also enables the accommodation and dispersion of more of it. After the loading of K_2_CO_3_, there is sufficient space for CO_2_ diffusion to be captured by active sites. In contrast, the pore size of the spherical structured Al_2_O_3_-1 appears relatively narrower, and after K_2_CO_3_ loading, it is prone to blockage, leading to greater resistance to CO_2_ diffusion within its interior.

**Figure 6:**
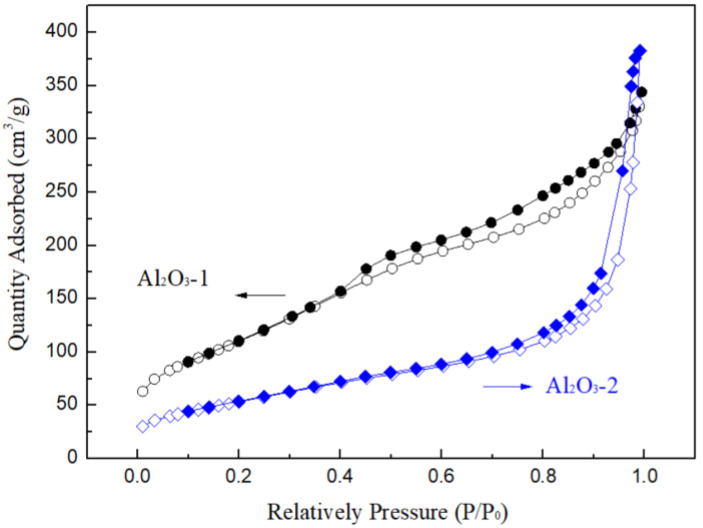
N_2_ adsorption (hollow) and desorption (solid) isotherms of Al_2_O_3_-1 and Al_2_O_3_-2.

The hierarchical structures of the two as-prepared Al_2_O_3_ samples were characterized using N_2_ adsorption–desorption isotherms, as shown in Figure 6. It is evident that the nitrogen adsorption isotherms for both materials have been classified as type IV according to the International Union of Pure and Applied Chemistry (IUPAC) [51], signifying the basic characteristic of mesoporous materials. The hysteresis loop exhibited by Al_2_O_3_-2 is typically characterized as type H1, which is a feature commonly observed in mesoporous materials with a relatively uniform pore size distribution. The weakly pronounced condensation steps, indicative of small mesopores, and the narrow hysteresis loop observed at high relative pressures reflect the textural larger pores that are formed between plate-like particles. It is documented in the literature that small mesopores, with diameters less than 4 nm, are formed between primary crystallites, while larger mesopores, with diameters greater than 20 nm, are formed between the secondary aggregated particles [52].

The Al_2_O_3_-1 curves display a type H3 hysteresis loop, characterized by the absence of a clear saturation adsorption platform. These curves indicate the presence of weak condensation steps, which are associated with the formation of larger slit-like mesopores between plate-like particles [45]. Additionally, a slightly wider hysteresis loop is observed within the relative pressure range of 0.4 to 1.0. The wider hysteresis loop is attributed to the irregularity of the pore structure. Generally, the width of the hysteresis loop is indicative of the connectivity between pores: a wider loop may suggest a lower degree of connectivity, while a narrower loop indicates a higher degree of connectivity between the pores. Obviously, the superior connectivity of the pore structure in Al_2_O_3_-2 is more favorable for the loading of K_2_CO_3_ solution. Super connectivity allows for the uniform dispersion of K_2_CO_3_ within the pore channels, preventing channel blockage and, in turn, facilitating the diffusion of CO_2_. The enhanced connectivity also promotes contact with a greater number of active sites, thereby increasing the adsorption capacity for CO_2_.

The pore structure parameters of the samples, including specific surface area, pore volume, and average pore size, are listed in Table 1. It can see that Al_2_O_3_-1 exhibits a higher BET surface area; however, this does not necessarily imply that more active sites would be exposed to CO_2_ after K_2_CO_3_ loading. The relatively small average pore diameter may lead to significant pore blockage, resulting in the loss of a large number of active sites. In contrast, Al_2_O_3_-2, which has a similar total pore volume to Al_2_O_3_-1, exhibits a much larger average pore diameter and better pore connectivity, as inferred from Figure 6. Consequently, this facilitates the uniform dispersion of K_2_CO_3_, the exposure of alkaline active sites, and the reduction of CO_2_ diffusion resistance, thereby further enhancing the utilization rate of active sites and the CO_2_ adsorption capacity.

**Figure 7:**
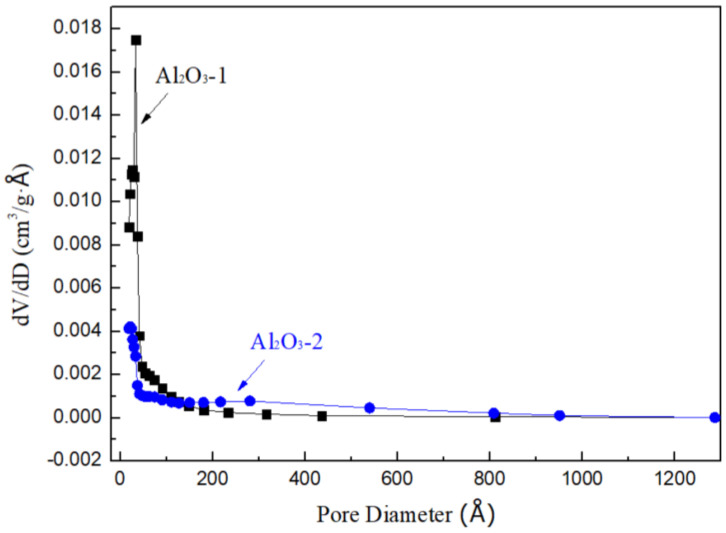
Pore size distributions of Al_2_O_3_-1 and Al_2_O_3_-2.

Figure 7 reveals that Al_2_O_3_-1 has a higher BET surface area and a smaller average pore diameter due to its abundance of micropores, whereas Al_2_O_3_-2, with a greater number of mesopores and macropores, has a lower BET surface area and a larger average pore diameter.

**Figure 8:**
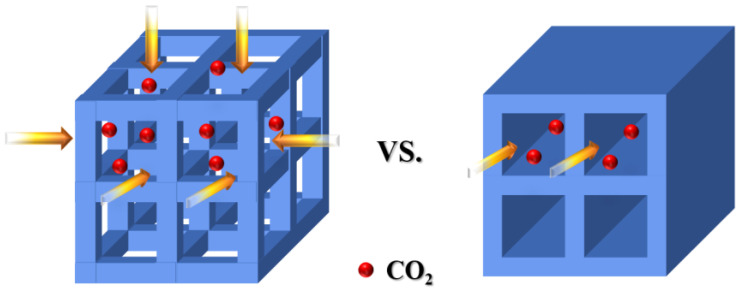
A comparative schematic of CO_2_ diffusion in interconnected and non-interconnected pore channels. The arrow represents the direction of molecular diffusion.

In summary, the connectivity of the pores and the large pore diameter of the support are deduced as key factors for achieving high CO_2_ adsorption capacity. The comparative schematic of CO_2_ diffusion in the interconnected and non-interconnected pore channels is depicted in Figure 8. It is evident that the interconnected pores significantly facilitate the diffusion of CO_2_.

**Figure 9:**
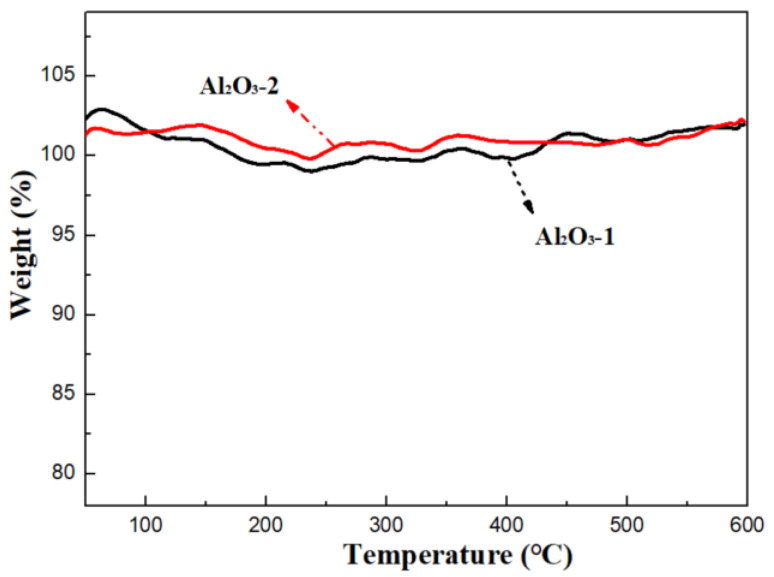
TGA curves of Al_2_O_3_-1 and Al_2_O_3_-2.

The thermal stability of Al_2_O_3_-1 and Al_2_O_3_-2 was examined using TGA, as depicted in Figure 9. Neither of the two aluminum oxide samples exhibited weight loss or endothermic peaks throughout the temperature-programmed process, suggesting that the aluminum oxide support is thermally stable at the adsorption and desorption temperatures of 25 °C and 350 °C. Consequently, the K_2_CO_3_/Al_2_O_3_ sorbents have the potential to be reused multiple times without losing amine sites.

### 3.3. Recycling Performance

**Figure 10:**
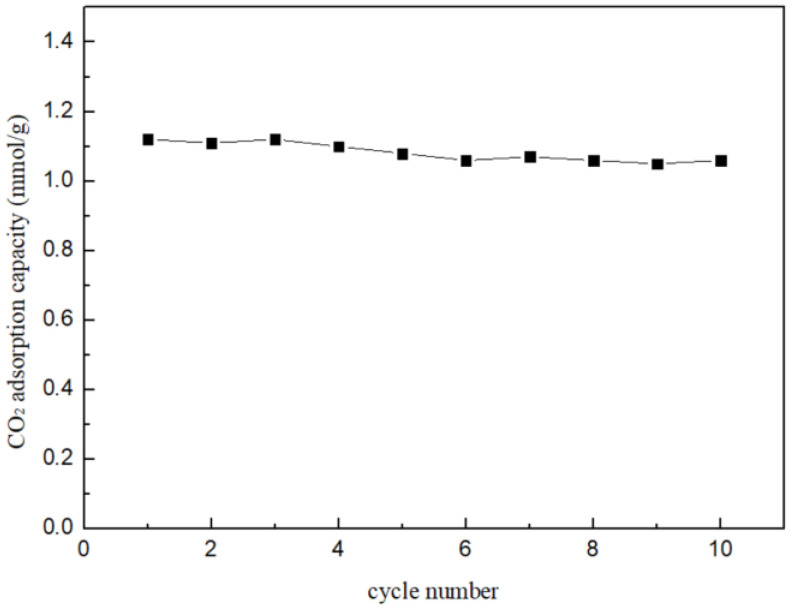
Recycling performance of 20% K_2_CO_3_/Al_2_O_3_-2 adsorbent after 10 cycles.

The regeneration performance of the adsorbent was evaluated and is depicted in Figure 10. Typically, after the CO_2_ sorption process in a closed-loop environmental control system, adsorbents are regenerated by increasing the temperature or introducing high-temperature steam. In our experiment, the used adsorbents were regenerated at 350 °C, a temperature at which the reacted products can be completely regenerated [46]. After 10 cycles, the CO_2_ adsorption capacity of the 20% K_2_CO_3_/Al_2_O_3_ sorbent showed virtually no decline, demonstrating that the prepared sorbent can be reused repeatedly while maintaining its CO_2_ adsorption capacity.

## 4. Conclusions

A series of K_2_CO_3_-based adsorbents were synthesized in this work. The CO_2_ sorption performance was investigated in detail under ambient temperature conditions and at a CO_2_ concentration of 10% using a fixed-bed flow sorption system, equipped with gas flow controllers and an integrated online gas chromatograph system. A 20 wt.% loading of K_2_CO_3_ was impregnated onto various supports, including Al_2_O_3_, zeolites ZSM-5, MCM-41, 5A, β, and NaY, CSAC, and CAC. Among these supports, Al_2_O_3_ was identified as the superior support, leading to further optimization of its pore structure by varying the preparation conditions to enhance CO_2_ adsorption capacity. The sorbent of 20% K_2_CO_3_/Al_2_O_3_-2 achieved the maximum CO_2_ adsorption capacity of 1.12 mmol g^−1^, and the utilization rate of K_2_CO_3_ was 77%. The pore volume and pore size distribution were found to significantly influence CO_2_ adsorption, as larger, interconnected pores enabled the uniform dispersion of the alkaline active sites, thereby enhancing the utilization of K_2_CO_3_. Moreover, the optimum adsorbent demonstrated full regenerability at 350 °C, highlighting its excellent recyclability. The K_2_CO_3_/Al_2_O_3_ sorbent may be an excellent choice for CO_2_ removal in coal-fired power plants.

## Figures and Tables

**Table 1:** Textual characteristics of Al_2_O_3_-1 and Al_2_O_3_-2.

Preparation Method	BET Surface Area (m^2^ g^−1^)	Pore Volume (cm^3^ g^−1^)	Average Pore Diameter (nm)
Al_2_O_3_-1	417	0.53	4.9
Al_2_O_3_-2	196	0.59	11.89

## Data Availability

Data are available on request due to restrictions.
